# Minimally interrupted novel oral anticoagulant versus uninterrupted vitamin K antagonist during atrial fibrillation ablation

**DOI:** 10.1007/s10840-018-0417-0

**Published:** 2018-08-03

**Authors:** John De Heide, Christiaan J. Vroegh, Rohit E. Bhagwandien, Sip A. Wijchers, Tamas Szili-Torok, Felix Zijlstra, Mattie J. Lenzen, S. C. Yap

**Affiliations:** Department of Cardiology, Erasmus Medical Center, P.O. Box 2040, 3000 CA Rotterdam, the Netherlands

**Keywords:** Atrial fibrillation, Novel oral anticoagulation, Vitamin-K antagonist, Catheter ablation, Bleeding, Stroke

## Abstract

**Purpose:**

The safety and efficacy of a minimally interrupted novel oral anticoagulant (NOAC) strategy at the time of atrial fibrillation (AF) ablation is uncertain. The purpose of this study was to compare rates of bleeding and thromboembolic events between minimally interrupted NOAC and uninterrupted vitamin K antagonist (VKA) in patients undergoing AF ablation.

**Methods:**

This was a retrospective single-center cohort study of consecutive patients who underwent AF catheter ablation between January 2013 and April 2017. Endpoints included major bleeding, clinically relevant non-major bleeding and systemic thromboembolic event from the time of ablation through 30 days. Bleeding events were defined by the Bleeding Academic Research Consortium (BARC) and International Society on Thrombosis and Haemostasis (ISTH).

**Results:**

A total of 637 patients were included in the analysis, 520 patients used uninterrupted VKA and 117 patients minimally interrupted NOAC (dabigatran: *n* = 68; apixaban: *n* = 30; rivaroxaban, *n* = 14; edoxaban, *n* = 5). The rate of clinically relevant non-major bleeding was lower in the NOAC group in comparison to the VKA group (BARC type 2: 2.6% versus 8.3%, *P* = 0.03; ISTH: 0% versus 3.8%, *P* = 0.03). Rates of major bleeding were similar between groups (BARC type 3 to 5: 3.4% versus 4.2%, *P* = NS; ISTH: 6.0% versus 8.7%, *P* = NS; for NOAC and VKA groups, respectively). Rates of systemic embolism were 0% with minimally interrupted NOAC, and 0.6% with uninterrupted VKA (*P* = NS).

**Conclusions:**

In patients undergoing AF ablation, anticoagulation with minimally interrupted NOAC was associated with fewer clinically relevant non-major bleeding events in comparison with uninterrupted VKA without compromising thromboembolic safety.

## Introduction

Catheter ablation is increasingly used for the treatment of symptomatic atrial fibrillation (AF). Although catheter ablation of AF is considered safe, it may be associated with a low risk of stroke. One of the strategies to reduce this risk is to perform AF ablation with continuous oral anticoagulation. This strategy has been shown to be safe and effective with vitamin K antagonists (VKAs) [1]. However, there is an increased use of novel oral anticoagulants (NOACs) in the current AF population undergoing catheter ablation. NOACs have several advantages, including a rapid onset of therapeutic range of anticoagulation, predictability of the anticoagulant effect, and relatively short time to reversal of anticoagulation when the medication is withheld [2]. Several observational and randomized controlled trials (RCTs) have demonstrated that uninterrupted NOAC is as safe and effective in comparison to uninterrupted VKA in patients undergoing AF ablation [3–13]. A recent meta-analysis demonstrated that NOAC was even associated with less major bleeding compared with VKA in pooled RCTs [14]. The 2016 ESC guidelines give a class IIa indication to perform AF ablation with continuous oral anticoagulation with either VKA or NOAC [15].

However, the uninterrupted NOAC strategy does not reflect current clinical practice as most centers still use a minimally interrupted NOAC strategy [16]. There is limited data demonstrating the safety and efficacy of a minimally interrupted NOAC strategy. The aim of the present study was to compare the incidence of bleeding and thromboembolic complications of minimally interrupted NOAC versus uninterrupted VKA in patients undergoing catheter ablation of AF.

## Methods

### Study population

We evaluated consecutive patients who underwent catheter ablation of AF from January 2013 to April 2017 in the Erasmus Medical Center, Rotterdam, the Netherlands. We included patients with 2 specific anticoagulation regimens. The first group included patients who used periprocedural uninterrupted VKA (either acenocoumarol or marcoumar). The strategy of uninterrupted VKA was introduced in our institution at the end of 2012. The second group included patients who used periprocedural minimally interrupted NOAC (1 or 2 doses withheld). In February 2013, our first patient underwent catheter ablation using a minimally interrupted NOAC strategy. Patients who did not use oral anticoagulation and were accepted for catheter ablation of AF usually received a NOAC.

### Pre- and periprocedural protocol

All patients received therapeutic oral anticoagulation for at least 3 weeks prior to ablation. In patients using VKA the target INR level at the day of the procedure was 2.0 to 2.5. In patients using NOACs, anticoagulation was withheld for 24 h before the procedure (1 or 2 doses withheld). A cardiac CT was routinely performed weeks to months prior to ablation. CT imaging was mainly used to assess PV anatomy. Rarely, a left atrial thrombus could be found as an incidental finding. A preprocedural transesophageal echocardiogram was routinely performed on the same day or 1 day prior to ablation to exclude left atrial appendage (LAA) thrombus. In the case of LAA thrombus the procedure was canceled or postponed. During the procedure, a bolus of heparin was administered after sheath placement. Furthermore, immediately after transseptal puncture another bolus of heparin was given and a continuous heparin pump was started and adjusted to maintain an ACT of at least 300 s. We did not administer protamine routinely at the end of the procedure.

### Postprocedural protocol

VKA patients, who had an INR 2.0 or greater at the day of the procedure, continued their anticoagulation regimen with a target INR level of 2.0–3.0. VKA patients who had an INR below 2.0 at the day of the procedure were bridged with intravenous UFH for 24 h (starting 2 h after removal of sheaths). After these 24 h they received low molecular weight heparin until their INR level was equal or above 2.0. NOAC patients restarted NOAC in the evening of the procedure. Patients continued their oral anticoagulation for at least 3 months after the procedure.

### Study endpoints

Primary bleeding endpoints were major bleeding (within 30 days) as defined by the Bleeding Academic Research Consortium (BARC) and International Society on Thrombosis and Haemostasis (ISTH) [17, 18]. The reason to choose both classifications is that clinical trials reporting major bleeding either use ISTH and/or BARC classification. In our study, BARC types 3 to 5 were considered a major bleeding. Secondary bleeding endpoints were the individual BARC bleeding types (types 2, 3a, 3b, 3c, 5), clinically relevant non-major bleeding (CRNMB) according to ISTH [19], and any clinically relevant bleeding (BARC types 2 to 5; ISTH major bleeding and CRNMB). BARC type 2 bleeding most closely aligns with the ISTH CRNMB [19].

The primary thromboembolic endpoint was a composite of stroke, transient ischemic attack (TIA), or other systemic embolism within 30 days.

### Statistical analysis

Continuous parameters are presented as the mean ± SD as they were normally distributed. Categorical data are presented as frequencies and percentages. Comparisons between groups were performed with an independent Student *t* test, chi-square tests, or Fisher exact test. A *P*-value < 0.05 was considered statistically significant. Statistical analyses were performed using SPSS software (SPSS, version 21; IBM, Chicago, Illinois).

## Results

**Fig. 1:**
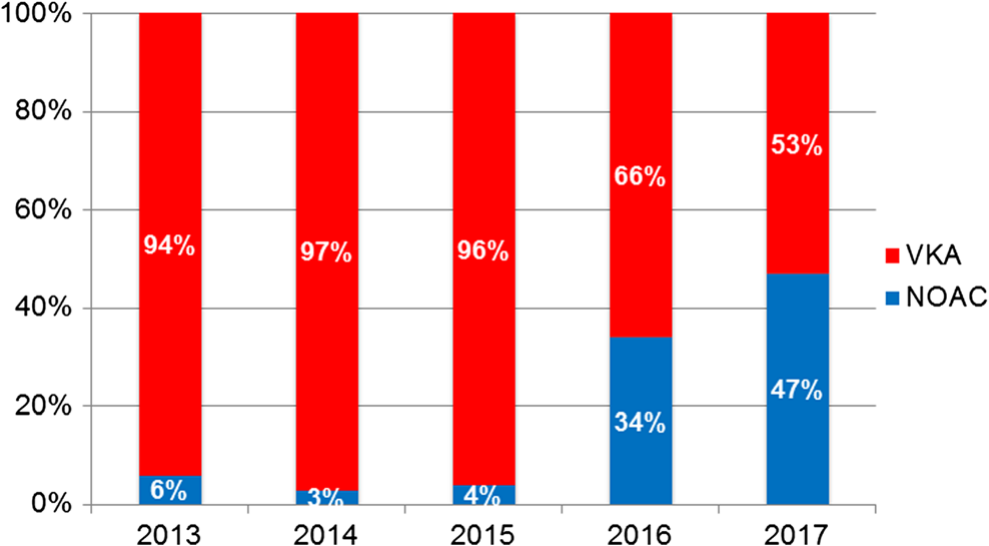
Proportion of periprocedural NOAC and VKA use over the years

A total of 637 patients (mean age 60 ± 9 years, 69% male) were included in the analysis, 520 patients (82%) used uninterrupted VKAs and 117 patients (18%) had a minimally interrupted NOAC strategy. In the NOAC group, the following NOACs were used: dabigatran (*n* = 68), apixaban (*n* = 30), rivaroxaban (*n* = 14), and edoxaban (*n* = 5). The NOAC group comprised more patients with long-standing persistent AF and a lower proportion of patients with a CHA_2_DS_2_-VASc ≥ 2 (Table 1). All other baseline variables were similar between groups. Figure 1 demonstrates the increased use of NOAC over the years in our AF ablation population.

**Table 1:** Baseline characteristics

Characteristic	Uninterrupted	Interrupted	*P*-value
	VKA	NOAC	
	*N* = 520	*N* = 117	
Age (years), mean ± SD	60 ± 10	60 ± 9	0.55
Male sex, n (%)	354 (68)	84 (72)	0.43
Atrial fibrillation, n (%):			0.048
Paroxysmal	392 (76)	86 (74)	
Persistent	116 (22)	24 (20)	
Long-standing persistent	10 (2)	7 (6)	
Hypertension	217 (42)	44 (38)	0.41
Diabetes mellitus	52 (10)	5 (4)	0.05
Coronary artery disease	62 (12)	7 (6)	0.06
Congestive heart failure	20 (4)	2 (2)	0.25
Left ventricular dysfunction	18 (3)	5 (4)	0.58
LA diameter (mm), mean ± SD	42 ± 6	43 ± 7	0.56
CHA_2_DS_2_-VASc score ≥ 2, n (%)	245 (47)	40 (34)	0.02
HAS-BLED score ≥ 3, n (%)	31 (6)	4 (3)	0.30
Body mass index, mean ± SD (kg/m^2^)	27.7 ± 4.1	27.2 ± 3.3	0.23
Technique of catheter ablation, n (%):			0.09
Cryoballoon	100 (19)	33 (28)	
Radiofrequency	402 (78)	83 (71)	
Laser	18 (3)	1 (1)	

LA = left atrium, NOAC = novel oral anticoagulant, VKA = vitamin K antagonist

### Bleeding complications

The rates of major bleeding, either by BARC or ISTH criteria, were similar between groups (Table 2). The rate of any clinically relevant bleeding (BARC types 2–5; composite of ISTH major bleeding and CRNMB) was lower with NOACs compared with VKAs. This difference was mainly due to a difference in clinically relevant non-major bleeding (CRNMB or BARC type 2) (Table 2). No patient in either group had a BARC type 3c (i.e., intracranial bleeding) or type 5 bleeding (i.e., fatal bleeding). Cardiac tamponade occurred in 4 patients (0.8%) of the VKA group and in 1 patient (0.9%) of the NOAC group (*P* = 1.00).

**Table 2:** Primary and secondary end points

	Uninterrupted	Interrupted	*P*-value
	VKA	NOAC	
	N = 520	N = 117	
Primary bleeding endpoints			
BARC 3–5 bleeding, n (%)	22 (4.2)	4 (3.4)	0.70
ISTH major bleeding, n (%)	45 (8.7)	7 (6.0)	0.34
Secondary bleeding endpoints			
Bleeding requiring medical attention that does not fit the criteria for types 3–5 (BARC 2), n (%)	43 (8.3)	3 (2.6)	0.03
Bleeding with hemoglobin drop of 30 to < 50 g/L or requiring transfusion (BARC 3a), n (%)	10 (1.9)	3 (2.6)	0.72
Bleeding with hemoglobin drop of ≥ 50 g/L, or requiring surgery or iv vasoactive agents, or cardiac tamponade (BARC 3b), n (%)	12 (2.3)	1 (0.9)	0.48
BARC 2–5 bleeding, n (%)	65 (12.5)	7 (6.0)	0.04
CRNMB, n (%)	20 (3.8)	–	0.03
ISTH major bleeding and CRNMB, n (%)	65 (12.5)	7 (6.0)	0.04
Primary thromboembolic endpoint			
Stroke, TIA, or other systemic embolism, n (%)	3 (0.6)	–	1.00

BARC = Bleeding Academic Research Consortium, CRNMB = clinically relevant non-major bleeding, ISTH = International Society on Thrombosis and Haemostasis, NOAC= novel oral anticoagulant, TIA = transient ischemic attack, VKA = vitamin K antagonist

### Thromboembolic complications

There were no differences in the systemic thromboembolic event rates between both groups (0.6% versus 0%, *P* = 1.00) (Table 2). In the VKA group, 1 patient (0.2%) experienced a vertebrobasilar stroke 3 days after the procedure. Three months after the procedure, this patient had a modified Rankin score of 1. Furthermore, 2 patients (0.4%) in the VKA group experienced a TIA 1 day after the procedure. They had an uneventful recovery. No patient in the NOAC group experienced a systemic thromboembolic event. No deaths occurred.

## Discussion

The main findings of our study are that (1) the rate of clinically relevant non-major bleeding was lower in patients with a minimally interrupted NOAC strategy compared with those with an uninterrupted VKA strategy, and (2) the rates of major bleeding and thromboembolic events were similar between groups.

Uninterrupted use of vitamin K antagonists (VKA) as periprocedural anticoagulant is currently widely accepted for patients undergoing catheter ablation of AF who are using VKA. However, there is an increased use of NOACs in the current AF ablation population. Despite initial concerns on the safety of using periprocedural NOAC [20], nowadays, several large RCTs have demonstrated the safety and efficacy of uninterrupted use of NOACs (i.e., dabigatran, rivaroxaban, apixaban) during AF ablation [5, 6, 12] (Table 3).

**Table 3:** Overview of major bleeding and thromboembolic events in large randomized controlled trials comparing periprocedural NOAC and VKA in patients undergoing catheter ablation of AF

Trial	BARC 3–5 bleedings	ISTH major bleeding	Thrombo-embolic events
RE-CIRCUIT [5] – VKA, N = 318	NA	6.9%	0.3%
RE-CIRCUIT [5] – uninterrupted dabigatran, *N* = 317	NA	1.6%*	0.0%
VENTURE-AF [6] – VKA, *N* = 124	NA	0.8%	0.8%
VENTURE-AF [6] – uninterrupted rivaroxaban, N = 124	NA	0.0%	0.0%
AXAFA [12] – VKA, *N* = 315	4.1%	4.4%	0.0%
AXAFA [12] – uninterrupted apixaban, *N* = 318	2.5%	3.1%	0.6%
ABRIDGE-J [13] – VKA, *N* = 222	NA	5.0%	0.5%
ABRIDGE-J [13] – interrupted dabigatran, *N* = 220	NA	1.4%*	0.0%
AEIOU [21] – uninterrupted apixaban, *N* = 150	1.3%	NA	0.7%
AEIOU [21] – interrupted apixaban, *N* = 145	2.1%	NA	0.7%

*Statistically significant difference in comparison to the VKA group. BARC = Bleeding Academic Research Consortium, ISTH = International Society on Thrombosis and Haemostasis, NA = not available, NOAC = novel oral anticoagulant, TIA = transient ischemic attack, VKA = vitamin K antagonist

In clinical practice, however, most centers still use a minimally interrupted NOAC strategy [16]. The European Snapshot Survey on Procedural Routines in Atrial Fibrillation Ablation (ESS-PRAFA) in 2015 demonstrated that AF ablations were performed with a minimally interrupted NOAC strategy (1–2 doses withheld) in 53% of procedures, interrupted NOAC ≥2 days in 34%, and an uninterrupted NOAC strategy in 14% [16]. The ABlation peRIoperative DabiGatran in use Envisioning in Japan (ABRIDGE-J) randomized trial demonstrated that anticoagulation with minimally interrupted dabigatran (1 or 2 doses withheld) was associated with fewer ISTH major bleeding complications than uninterrupted VKA with no increase in thromboembolic events (Table 3) [13]. In addition, the Apixaban Evaluation of Interrupted Or Uninterrupted anticoagulation for ablation of atrial fibrillation (AEIOU) randomized trial showed no difference between continuous apixaban compared with minimally interrupted apixaban (1 dose withheld) with regard to major bleeding (BARC 3–5) or thromboembolic events (Table 3) [21]. Finally, a recent meta-analysis of 4 randomized and 9 prospective observational studies (*N* = 5463) found that minimally interrupted and continuous NOAC strategy were both safe and non-inferior strategies compared with uninterrupted VKA [14]. Our study extends on these results demonstrating less clinically relevant non-major bleeding events with minimally interrupted NOAC in comparison with uninterrupted VKA without compromising thromboembolic safety.

One of the reasons to choose an uninterrupted NOAC strategy instead of a minimally interrupted NOAC strategy is to maximally reduce the incidence of thromboembolic events. However, the risk of a systemic thromboembolic event using a minimally interrupted NOAC strategy is already low (< 0.7%) [13, 14, 21]. Furthermore, continuous anticoagulation does not prevent all acute brain lesions, which can be caused by debris from ablation lesions, air emboli, or small thrombi [22]. This was demonstrated by the MRI substudy of the AXAFA trial in which acute brain lesions occurred in 27% of patients despite uninterrupted apixaban [12]. Further research is required to establish the optimal NOAC dosing strategy (minimally interrupted or uninterrupted) with regard to both bleeding and thromboembolic risk. Another question is whether every NOAC is effective in preventing periprocedural thromboembolic complications. RCTs with dabigatran (RE-CIRCUIT) and rivaroxaban (VENTURE-AF) did not show any thromboembolic events [5, 6], while RCTs with apixaban (AXAFA, AEIOU) showed a low thromboembolic event rate [12, 21].

### Study limitations

There were differences in baseline characteristics between the study groups. The VKA group had a higher proportion of patients with a CHA_2_DS_2_-VASc ≥ 2 in comparison to the NOAC group (47% versus 34%). This difference can be explained by the fact that in patients who did not use an oral anticoagulant (low CHA_2_DS_2_-VASc score) and were accepted for catheter ablation, a NOAC was preferentially started as periprocedural anticoagulation regime. This difference in CHA_2_DS_2_-VASc score could potentially lower the risk of thromboembolic and bleeding events in the NOAC group. Furthermore, patients used different NOACs in the present study. The limited number of NOAC patients precluded further subanalysis for the different NOACs.

## Conclusions

In patients undergoing catheter ablation of AF, a minimally interrupted NOAC strategy was associated with fewer clinically relevant non-major bleeding compared with uninterrupted VKA. The risk of major bleeding and thromboembolic events was similar between both strategies. Our study reinforces the safety and efficacy of a minimally interrupted NOAC strategy as periprocedural anticoagulant in patients undergoing catheter ablation of AF.

## References

[1] Di Biase L, Burkhardt JD, Santangeli P, Mohanty P, Sanchez JE, Horton R (2014). Periprocedural stroke and bleeding complications in patients undergoing catheter ablation of atrial fibrillation with different anticoagulation management: results from the role of coumadin in preventing thromboembolism in atrial fibrillation (AF) patients undergoing catheter ablation (COMPARE) randomized trial. Circulation.

[2] Jackson LR, Becker RC (2014). Novel oral anticoagulants: pharmacology, coagulation measures, and considerations for reversal. J Thromb Thrombolysis.

[3] Di Biase L, Lakkireddy D, Trivedi C, Deneke T, Martinek M, Mohanty S (2015). Feasibility and safety of uninterrupted periprocedural apixaban administration in patients undergoing radiofrequency catheter ablation for atrial fibrillation: results from a multicenter study. Heart Rhythm.

[4] Aryal MR, Ukaigwe A, Pandit A, Karmacharya P, Pradhan R, Mainali NR, Pathak R, Jalota L, Bhandari Y, Donato A (2014). Meta-analysis of efficacy and safety of rivaroxaban compared with warfarin or dabigatran in patients undergoing catheter ablation for atrial fibrillation. Am J Cardiol.

[5] Calkins H, Willems S, Gerstenfeld EP, Verma A, Schilling R, Hohnloser SH, Okumura K, Serota H, Nordaby M, Guiver K, Biss B, Brouwer MA, Grimaldi M, RE-CIRCUIT Investigators (2017). Uninterrupted dabigatran versus warfarin for ablation in atrial fibrillation. N Engl J Med.

[6] Cappato R, Marchlinski FE, Hohnloser SH, Naccarelli GV, Xiang J, Wilber DJ, Ma CS, Hess S, Wells DS, Juang G, Vijgen J, Hügl BJ, Balasubramaniam R, de Chillou C, Davies DW, Fields LE, Natale A, VENTURE-AF Investigators (2015). Uninterrupted rivaroxaban vs. uninterrupted vitamin K antagonists for catheter ablation in non-valvular atrial fibrillation. Eur Heart J.

[7] Hohnloser SH, Camm AJ (2013). Safety and efficacy of dabigatran etexilate during catheter ablation of atrial fibrillation: a meta-analysis of the literature. Europace.

[8] Kaess BM, Ammar S, Reents T, Dillier R, Lennerz C, Semmler V, Grebmer C, Bourier F, Buiatti A, Kolb C, Deisenhofer I, Hessling G (2015). Comparison of safety of left atrial catheter ablation procedures for atrial arrhythmias under continuous anticoagulation with apixaban versus phenprocoumon. Am J Cardiol.

[9] Lakkireddy D, Reddy YM, Di Biase L, Vallakati A, Mansour MC, Santangeli P (2014). Feasibility and safety of uninterrupted rivaroxaban for periprocedural anticoagulation in patients undergoing radiofrequency ablation for atrial fibrillation: results from a multicenter prospective registry. J Am Coll Cardiol.

[10] Santarpia G, De Rosa S, Polimeni A, Giampa S, Micieli M, Curcio A (2015). Efficacy and safety of non-vitamin K antagonist oral anticoagulants versus vitamin K antagonist oral anticoagulants in patients undergoing radiofrequency catheter ablation of atrial fibrillation: a meta-analysis. PLoS One.

[11] Wu S, Yang YM, Zhu J, Wan HB, Wang J, Zhang H, Shao XH (2016). Meta-analysis of efficacy and safety of new oral anticoagulants compared with uninterrupted vitamin K antagonists in patients undergoing catheter ablation for atrial fibrillation. Am J Cardiol.

[12] Kirchhof P, Haeusler KG, Blank B, De Bono J, Callans D, Elvan A et al. Apixaban in patients at risk of stroke undergoing atrial fibrillation ablation. Eur Heart J. 2018. 10.1093/eurheartj/ehy176. 10.1093/eurheartj/ehy176 PMC6110196 29579168

[13] Nogami A, Machino T, Harada T, Nakano Y, Yoshida Y, Goya M (2017). Clinical benefit of minimally-interrupted dabigatran versus uninterrupted warfarin for catheter ablation of atrial fibrillation: a prospective randomized multicenter trial.

[14] Ha FJ, Barra S, Brown AJ, Begley DA, Grace AA, Agarwal S (2018). Continuous and minimally-interrupted direct oral anticoagulant are both safe compared with vitamin K antagonist for atrial fibrillation ablation: an updated meta-analysis. Int J Cardiol.

[15] Kirchhof P, Benussi S, Kotecha D, Ahlsson A, Atar D, Casadei B, Castella M, Diener HC, Heidbuchel H, Hendriks J, Hindricks G, Manolis AS, Oldgren J, Popescu BA, Schotten U, van Putte B, Vardas P, Agewall S, Camm J, Baron Esquivias G, Budts W, Carerj S, Casselman F, Coca A, de Caterina R, Deftereos S, Dobrev D, Ferro JM, Filippatos G, Fitzsimons D, Gorenek B, Guenoun M, Hohnloser SH, Kolh P, Lip GYH, Manolis A, McMurray J, Ponikowski P, Rosenhek R, Ruschitzka F, Savelieva I, Sharma S, Suwalski P, Tamargo JL, Taylor CJ, van Gelder IC, Voors AA, Windecker S, Zamorano JL, Zeppenfeld K (2016). 2016 ESC guidelines for the management of atrial fibrillation developed in collaboration with EACTS. Eur Heart J.

[16] Potpara TS, Larsen TB, Deharo JC, Rossvoll O, Dagres N, Todd D, Pison L, Proclemer A, Purefellner H, Blomstrom-Lundqvist C, Blomstrom-Lundqvist C, Bongiorni MG, Chen J, Dagres N, Estner H, Hernandez-Madrid A, Hocini M, Larsen TB, Pison L, Potpara T, Proclemer A, Sciraffia E, Todd D, Scientific Initiatives Committee of the Euro (2015). Oral anticoagulant therapy for stroke prevention in patients with atrial fibrillation undergoing ablation: results from the first European snapshot survey on procedural routines for atrial fibrillation ablation (ESS-PRAFA). Europace.

[17] Mehran R, Rao SV, Bhatt DL, Gibson CM, Caixeta A, Eikelboom J, Kaul S, Wiviott SD, Menon V, Nikolsky E, Serebruany V, Valgimigli M, Vranckx P, Taggart D, Sabik JF, Cutlip DE, Krucoff MW, Ohman EM, Steg PG, White H (2011). Standardized bleeding definitions for cardiovascular clinical trials: a consensus report from the bleeding academic research consortium. Circulation.

[18] Schulman S, Kearon C, Subcommittee on Control of Anticoagulation of the Scientific, Standardization Committee of the International Society on Thrombosis and Haemostasis (2005). Definition of major bleeding in clinical investigations of antihemostatic medicinal products in non-surgical patients. J Thromb Haemost.

[19] Kaatz S, Ahmad D, Spyropoulos AC, Schulman S, Subcommittee on Control of Anticoagulation (2015). Definition of clinically relevant non-major bleeding in studies of anticoagulants in atrial fibrillation and venous thromboembolic disease in non-surgical patients: communication from the SSC of the ISTH. J Thromb Haemost.

[20] Lakkireddy D, Reddy YM, Di Biase L, Vanga SR, Santangeli P, Swarup V (2012). Feasibility and safety of dabigatran versus warfarin for periprocedural anticoagulation in patients undergoing radiofrequency ablation for atrial fibrillation: results from a multicenter prospective registry. J Am Coll Cardiol.

[21] Reynolds MR, Scott Allison J, Natale A, Weisberg IL, Ellenbogen KA, Richards M et al. A prospective randomized trial of apixaban dosing during atrial fibrillation ablation. JACC Clin Electrophysiol. 2018;4(5):580–8. 10.1016/j.jacep.2017.11.005 29798783

[22] Takami M, Lehmann HI, Parker KD, Welker KM, Johnson SB, Packer DL (2016). Effect of left atrial ablation process and strategy on microemboli formation during irrigated radiofrequency catheter ablation in an *in vivo* model. Circ Arrhythm Electrophysiol.

